# Excessive Tearing and Lacrimal and Canalicular Blockage Secondary to Docetaxel in Breast Cancer Patients in the Metastatic and Adjuvant Settings

**DOI:** 10.3390/curroncol33060359

**Published:** 2026-06-15

**Authors:** Margaret L. Pfeiffer, Bita Esmaeli

**Affiliations:** 1 Key-Whitman Eye Center, Dallas, TX 75243, USA; 2 University of Texas Southwestern Medical Center, Dallas, TX 75390, USA; 3 Department of Ophthalmology, Houston Methodist Academic Institute, Houston, TX 77030, USA; 4 Department of Clinical Sciences, University of Houston Tilman J. Fertitta Family College of Medicine, Houston, TX 77001, USA; 5 Oncologic Ophthalmic Plastic & Orbital Surgery, Mann Eye Institute, Houston, TX 77005, USA

**Keywords:** epiphora, tearing, docetaxel, canalicular stenosis, punctal stenosis, lacrimal duct blockage, breast cancer

## Abstract

While most ophthalmologists and medical oncologists are aware of the excessive tearing and canalicular and lacrimal duct blockage associated with docetaxel, there is lack of deeper understanding of the appropriate management of this side effect and what clinical settings justify surgical intervention as opposed to conservative management. In this review, we summarize the findings in the seminal original research studies that document the association between the frequency of administration of docetaxel and treatment duration and the frequency of excessive tearing as a subjective symptom versus canalicular and lacrimal duct blockage as anatomic findings seen during probing and irrigation. Based on the published literature to date, we note that excessive tearing is a common and important side effect of docetaxel and can be seen in breast cancer patients who are receiving docetaxel either weekly or every three weeks in metastatic or adjuvant settings. However, the anatomic findings of canalicular and lacrimal duct stenosis are almost exclusively seen in patients receiving weekly docetaxel or in patients with metastatic breast cancer who are treated with docetaxel for prolonged periods. Lacrimal duct blockage is much less commonly reported in patients with early breast cancer who are receiving docetaxel every three weeks for short durations and, to our knowledge to date, have not been reported in the literature in breast cancer patients receiving docetaxel in the adjuvant setting.

## Simple Summary

Excessive tearing is a common and important side effect of docetaxel and can be observed in breast cancer patients receiving docetaxel in the adjuvant setting or for treatment of metastatic disease. However, the anatomic findings of canalicular and lacrimal duct blockage are essentially observed only in patients treated with a weekly administration of docetaxel or in patients with metastatic breast cancer who have had prolonged treatment with an every-3-weeks administration of docetaxel and not in patients with early stage breast cancer who receive every-3-weeks docetaxel for a few doses in the adjuvant setting.

## 1. Introduction

Excessive tearing (epiphora) is a relatively common symptom in cancer patients. The etiologies of excessive tearing in cancer patients are diverse and can be secondary to cancer treatments such as chemotherapy or radiation therapy [1,2]. Excessive tearing has been reported as a common side effect associated with docetaxel, a commonly used chemotherapy, and is most often seen after weekly administration or with prolonged use of docetaxel administered every three weeks [3,4,5,6,7,8,9,10,11,12]. Docetaxel is secreted into the tear film and can cause chronic inflammation and eventual fibrosis and blockage of the tear drainage system, starting with stenosis of the puncta and canaliculi and, in severe cases, extending to blockage of the nasolacrimal duct [4].

Although canalicular stenosis as an anatomic finding has been well documented in association with weekly administration of docetaxel, it is important to recognize that not all cases of tearing associated with docetaxel are due to canalicular stenosis. In fact, excessive tearing associated with docetaxel in its current frequency of administration in the United States, which is administration every 3 weeks, is likely more frequently due to tear film insufficiency and hypersecretion of tears as a reflex due to dry eye syndrome. Particularly in breast cancer patients, dry eye syndrome can be due to or exacerbated by a menopausal hormonal state or due to anti-estrogen hormonal therapy [1,2]. In head and neck cancer patients treated with docetaxel, radiation near the lacrimal gland and ocular surface can induce dry eyes. Dry eyes can also be caused by the impact of chemotherapy slowing down the proliferation of all glandular tissue including the meibomian glands of the eyelids and the major and accessory lacrimal glands that secrete different components of the tear film [13,14].

In this work, we will review the available literature on excessive tearing associated with docetaxel. We will distinguish the expected frequency and severity of this side effect in patients who receive docetaxel weekly versus every three weeks and highlight the significant difference in findings in patients who received docetaxel for metastatic breast cancer versus those who were exposed to short durations of docetaxel in the adjuvant setting. We also will provide practical advice for the treating providers, both ophthalmologists and medical oncologists.

We believe that understanding the circumstances where excessive tearing is associated with permanent anatomic changes in the tear drainage system is important for ophthalmologists to be able to offer timely surgical interventions. It is equally important to understand when excessive tearing associated with docetaxel is expected to be transient and may have an etiology other than canalicular stenosis. This latter understanding is particularly relevant for breast cancer patients who are receiving docetaxel in the adjuvant setting and can inform the providers so that unnecessary surgery can be avoided. These details may not be readily understood by the treating ophthalmologists and medical oncologists. This manuscript aims to bridge this gap in knowledge.

## 2. Materials and Methods

A PubMed search was carried out on 20 January 2026, for the terms “docetaxel and tearing,” “docetaxel and epiphora,” “docetaxel and canalicular stenosis,” “docetaxel and punctal stenosis,” and “docetaxel and lacrimal duct blockage.” Abstracts were reviewed for relevance, and full text articles and their references were reviewed in detail.

## 3. Background Information: Defining Excessive Tearing and Findings on the Eye Exam

The presence or absence of excessive tearing (epiphora) is subjective, and its diagnosis hinges primarily on patient-reported symptoms. Excessive tearing has been reported in up to 93% (28/30) [15] of cancer patients treated with docetaxel, but its frequency is highly dependent on the frequency of administration of the drug (weekly versus every three weeks) and duration of treatment [3,6,7,8,9,10,11]. Furthermore, not all cases of tearing are associated with the anatomic finding of stenosis or blockage of the tear drainage system (punctum, canaliculus, nasolacrimal duct).

Excessive tearing has a negative impact on quality of life [16]. Epiphora (defined as tears that run down the cheek) not only interferes with the quality of vision but is also bothersome as patients are often asked if they are emotionally crying, cannot wear makeup normally, and have irritated eyes and eyelid skin from the constant wiping of tears. Excessive tearing may be a unilateral or bilateral problem or may impact the two eyes asymmetrically depending on the etiology.

The evaluation of cancer patients with excessive tearing should include a thorough medical history with special attention to the timing of the onset of tearing, history of cancer, specific chemotherapy regimen including frequency of administration and duration of exposure, surgical history in the orbital and nasal area, and history of exposure to radiation and radiation dosage in the periocular area.

There is no definitive objective metric for the diagnosis of epiphora. Comprehensive clinical evaluation of cancer-associated excessive tearing includes, at a minimum, a slit lamp examination with attention to the ocular surface, position of the eyelids and puncta; fluorescein staining of the cornea to rule out dry eyes; and probing and irrigation of the tear drainage apparatus. Attention should be directed to active tearing (presence of epiphora defined as visible tear drops on the face) during examination. This could be a sign of a more significant underlying abnormality compared with patients who complain of subjective “tearing” but have no visible signs of excessive tears during exam. The size, shape, and position of the puncta should be evaluated. The position of the lower eyelid and any anatomic abnormalities in the medial canthus and lacrimal sac area should be noted. Probing and irrigation (“syringing”) is best performed by an ophthalmic plastic surgeon. Clinicians may additionally perform, according to their preferred practice patterns and findings, a dye disappearance test, Schirmer’s testing, nasal endoscopy, or a variety of other ancillary dry eye testing.

The primary aim of the clinical exam in patients with excessive tearing is to distinguish between its two primary causes: blockage of the tear drainage apparatus versus reflex tearing. Reflex tearing occurs when the eye produces more tears than normal in response to an external stimulant and is frequently caused by dry eyes or allergic conjunctivitis. In contrast, tear drainage abnormalities consist of punctal, canalicular, or nasolacrimal duct stenosis or obstruction, all of which prevent normal tear outflow from the ocular surface.

Probing and irrigation is a crucial step in the clinical evaluation of a tearing patient as it identifies anatomic changes in the tear drainage apparatus. This test requires experience to execute correctly. Each component of the tear drainage apparatus (punctum, canaliculus, or nasolacrimal duct) may become narrow (stenotic) or completely occluded (obstructed), and the degree and specific location of this compromise is important and can be detected on probing and irrigation. Clinicians can feel a stenotic punctum or canaliculus with the lacrimal probe. An obstructed punctum will not allow entry of the lacrimal probe or even a punctal dilator. An obstructed canaliculus will not allow passage of the lacrimal probe, and irrigation of fluid will result in complete reflux from the same canaliculus. In severe canalicular stenosis, probing and irrigation may cause bleeding and discomfort for the patient during the examination. An obstructed nasolacrimal duct will cause reflex from the fellow canaliculus.

Docetaxel has been associated with both reflex tearing and stenosis or obstruction but in different clinical settings. Therefore, appropriate treatment of docetaxel-associated tearing hinges on proper characterization of the cause, as reflex tearing can simply be treated medically with the administration of lubricating and other prescription drops, whereas significant stenosis is an anatomic change that may necessitate surgical intervention. Blockage of the lacrimal drainage apparatus has also been described in association with other chemotherapy drugs such as 5-fluorouracil and S-1 [12].

## 4. Results

### 4.1. Weekly Docetaxel for Metastatic Breast Cancer and Canalicular Stenosis

Excessive tearing and its anatomic correlation to punctal and canalicular stenosis associated with weekly docetaxel was first reported in 2001 [3], although “conjunctivitis” had been reported previously as an adverse event in some of the earlier reported phase II studies using docetaxel [17]. Table 1 summarizes the published literature on excessive tearing and lacrimal duct blockage associated with docetaxel.

The specific association between docetaxel and punctal and canalicular stenosis was first recognized and described by Esmaeli et al. in 2001 in patients with metastatic breast cancer receiving docetaxel weekly [3]. Subsequently, this association became well established through multiple retrospective and prospective studies by the same group [3,6,7,8,9,11] and was replicated by other groups [9,18]. The Food and Drug Administration (FDA) updated the label for docetaxel in 2001 to add the following side effects to the section on post-marketing experience: “conjunctivitis, lacrimation or lacrimation with or without conjunctivitis, excessive tearing which may be attributable to lacrimal duct obstruction” [19]. Under the section on patient counseling, the label recommends, “Advise patients that vision disturbances and excessive tearing are associated with TAXOTERE administration.”

Through the above-mentioned publications, it was recognized that the dosing and frequency of docetaxel therapy were important in terms of the risk of canalicular stenosis. A higher cumulative dose and more frequent administration of docetaxel were both associated with anatomic changes in the canaliculus [5,6,7]. Furthermore, more severe canalicular stenosis or obstruction was seen in patients treated with a higher cumulative dose of docetaxel as opposed to moderate canalicular stenosis in patients treated with a lower cumulative dose [6,7].

Investigators reporting on this side effect began to recognize that there was a clear distinction in the severity of anatomic findings in patients receiving weekly docetaxel versus treatment every 3 weeks: weekly treatment more frequently led to canalicular stenosis. Reported ranges of canalicular stenosis on weekly treatment are 33–100% (Table 1) [3,5,6,8,9,15,18].

In a retrospective study in 2002 of 36 patients with metastatic breast cancer, with 18 patients in each treatment group, 9/18 (50%) of those on weekly treatment had symptomatic excessive tearing and significant canalicular stenosis on probing and irrigation, and 8 of these needed surgery [5]. In contrast, only 2/18 (11%) of patients in the every-3-weeks treatment group reported transient excessive tearing, neither of whom had canalicular stenosis on exam.

The same team conducted a prospective randomized study published in 2006 that enrolled 28 patients with metastatic breast cancer receiving weekly docetaxel treatment and 28 patients receiving every-3-weeks treatment [8]. All patients had a baseline eye exam including probing and irrigation by Dr. Esmaeli and were closely followed every four to six weeks. The findings in this prospective trial confirmed the observations in prior retrospective reports. Of the patients receiving weekly docetaxel, 18/28 (64%) developed excessive tearing and 9/18 (50%) had canalicular stenosis, for which surgery was recommended. Excessive tearing was classified as moderate in five patients and severe in six. Nine patients improved with tobramycin–dexamethasone drops given for at least six weeks. Nine patients had worsening canalicular stenosis, for which surgery was recommended. Six patients followed through with surgery: two bilateral silicone stent placement, three bilateral dacryocystorhinostomy (DCR), and one unilateral DCR and contralateral silicone stent placement. In contrast, excessive tearing as a symptom and canalicular stenosis as an anatomic finding were less common and less severe in the every-3-weeks docetaxel treatment group. Of the patients, 11/28 (39%) developed excessive tearing and only 2/28 (7%) had canalicular stenosis and required surgery [8]. Excessive tearing was more likely to be mild in this group (nine patients). Excessive tearing resolved in most patients (9/11) after at least six weeks of tobramycin–dexamethasone drops. Two patients had persistent excessive tearing and grade 1 canalicular stenosis and underwent surgery (bilateral DCR). The greater frequency of more severe excessive tearing in the weekly docetaxel treatment arm compared to the every-3-weeks docetaxel arm as well as the greater frequency of recommended surgical intervention in the weekly group were both statistically significant differences (*p* = 0.00095 and *p* = 0.04, respectively). The greater frequency of excessive tearing as a symptom in the weekly group was not statistically significant.

Another study of 21 patients on weekly therapy noted that excessive tearing and canalicular stenosis developed in 7 patients (33%) [9]. Surgery was recommended in two patients and two patients discontinued treatment specifically due to excessive tearing. These authors concluded that excessive tearing was a “major dose-limiting toxicity of weekly docetaxel.”

**Table 1:** Reported frequencies of excessive tearing and stenosis in patients on weekly and every-3-weeks docetaxel.

Weekly Docetaxel Treatment				
Study	Patient Population	Frequency of Excessive Tearing	Frequency of Stenosis	Outcomes of Excessive Tearing
Esmaeli 2001 [3]	Metastatic breast cancer	100% (3/3) ^a^	100% (3/3)	All persisted after docetaxel discontinued; 1 declined surgery, 1 had partial improvement with punctoplasty, 1 resolved after stenting
Esmaeli 2002 [6]	Metastatic breast cancer	100% (14/14) ^a^	100% (14/14)	All 11 patients who underwent surgery had resolution; 3 declined surgery and tearing persisted
Esmaeli 2002 [5]	Metastatic breast cancer	77% (14/18)	50% (9/18)	All 8 patients who underwent surgery had complete or near complete resolution; 1 declined surgery
Esteva 2002 [15]	Metastatic breast cancer	93% (28/30)	40% (12/30)	Not reported
Esmaeli 2003 [7]	Breast cancer (majority metastatic), prostate cancer, lung cancer, and others	100% (71/71) ^a^	Not reported	97% (29/30) who underwent surgery had complete resolution or improvement; 1 surgery failed; 21 declined surgery; 20 had mild symptoms and mild stenosis and surgery was not recommended
Esmaeli 2006 [8]	Metastatic breast cancer	64% (18/28)	50% (9/18)	50% (9/18) resolved with antibiotic-steroid drops and probing and irrigation; 6 underwent surgery; 3 declined surgery
Kintzel 2006 [17]	Metastatic breast cancer	6–93% ^b^	Not reported	Not reported
Tsalic 2006 [9]	Breast cancer (majority metastatic), non-small-cell lung cancer, nasopharyngeal carcinoma	33% (7/21)	33% (7/21)	3 resolved; 3 had persistent symptoms (2 of these declined surgery); 1 died
Leyssens 2010 [18]	Breast cancer (majority metastatic), prostate cancer	43% (17/40 eyes)	45% (18/40 eyes)	Canalicular stenosis resolved in 9 eyes; 3 eyes developed new stenosis at last follow-up; all declined surgery
Van Eijk 2022 [20]	Metastatic breast cancer	13% (13/100)	Not reported	Not reported
**Every-3-Weeks Docetaxel Treatment**				
**Study**	**Patient Population**	**Frequency of** **Excessive Tearing**	**Frequency of Stenosis**	**Outcomes Regarding** **Excessive Tearing**
Esmaeli 2002 [5]	Metastatic breast cancer	11% (2/18)	0%	All resolved after antibiotic-steroid drops and probing and irrigation
Esmaeli 2003 [7]	Breast cancer (majority metastatic), prostate cancer, lung cancer, and others ^c^	100% (77/77) ^a^	Not reported	96% (74/77) resolved with antibiotic-steroid drops and probing and irrigation; 4% (3/77) resolved after surgery
Esmaeli 2006 [8]	Metastatic breast cancer	39% (11/28)	7% (2/28)	82% (9/11) resolved with antibiotic-steroid drops and probing and irrigation; 7% (2/28) resolved after surgery
Chan 2013 [10]	Stage II breast cancer	86% (86/100)	4% (4/100)	51% (51/100) resolved with artificial tears; 29% (29/100) had persistent mild, intermittent tearing
Noguchi 2016 [21]	Breast cancer	13% (6/48)	Not reported	All resolved
Kang 2017 [22]	Breast and prostate cancer	100% (10/10) ^a^	At least 90% (9/10) ^d^	All resolved after surgery
Noguchi 2020 [23]	Breast cancer, lung cancer, head and neck cancer, prostate cancer, and others	8% (7/89)	Not reported	All resolved on topical therapies
Van Eijk 2022 [20]	Metastatic breast cancer	3% (3/100)	Not reported	Not reported
Iwai 2025 [24]	Breast cancer	128/10,000 person-years	Not reported	Not reported

^a^ Presence of excessive tearing was an inclusion criteria for these retrospective studies; ^b^ this review article reported data from multiple studies; ^c^ although most patients in this report were on every-3-weeks treatment, it also included five patients on every-2-weeks treatment; ^d^ please see Section 4.2 for a discussion of these findings.

### 4.2. Every-3-Weeks Docetaxel for Metastatic Disease and in the Adjuvant Setting in Early Breast Cancer: Primarily Self-Limited Reflex Tearing

Every-3-weeks docetaxel administration in patients with metastatic and early breast cancer can cause a high rate of excessive tearing, ranging from 3 to 100%, but, fortunately, the anatomic finding of canalicular and lacrimal duct blockage is uncommon in this setting and usually resolves after the cessation of treatment and with conservative measures [5,7,8,10,20,21,22,23]. In addition to its popularity for the treatment of metastatic breast cancer, docetaxel is also commonly used in patients with early breast cancer in the adjuvant setting, meaning after surgery or localized radiation therapy, and usually in combination with other chemotherapy drugs. As adjuvant therapy, docetaxel is typically administered every three weeks.

Chan et al. conducted a prospective study of 100 patients, a majority with stage II breast cancer, on docetaxel-based chemotherapy administered every three weeks [10]. Most patients in this study also concurrently received other types of chemotherapy. These authors reported that 86 patients (86%) had excessive tearing. Patients reported excessive tearing as either mild or moderate, and 51 patients reported complete resolution of tearing by four months after the last cycle of chemotherapy. In total, 29 patients had persistent symptoms after docetaxel cessation and described only mild, intermittent tearing. Six patients had what the authors describe as worsening of nasolacrimal irrigation over the study period, and one developed complete obstruction. The authors did not specify if the anatomic abnormalities were at the level of the punctum, canaliculus, or nasolacrimal duct. This article’s results are confounded by the fact that 27 patients (27%) had baseline punctal stenosis and 17 patients (17%) had baseline lacrimal duct obstruction detected on probing and irrigation prior to the initiation of docetaxel.

Chan et al. concluded that excessive tearing secondary to docetaxel used as adjuvant therapy was prevalent yet typically mild and temporary, and thus, patients should be reassured that permanent symptoms in this clinical setting are uncommon and surgery is not necessary. These conclusions are in agreement with prior observations in patients with metastatic breast cancer on every-3-weeks treatment for short durations [5,7,8,11]. One should note that while there is a range of frequencies of excessive tearing in patients with metastatic breast cancer on every-3-weeks treatment (see Table 1), the frequency of stenosis is low overall. In the vast majority of patients, tearing resolved with drops and probing and irrigation [5,8,10,21,23].

In a report from South Korea that included a diverse group of cancer patients and cancer treatments associated with excessive tearing such as direct tumor extension into the lacrimal duct, radiation therapy, radioactive iodine therapy, S-1 and docetaxel, a small proportion of the cohort, 10 patients with either breast or prostate cancer, were evaluated for excessive tearing associated with every-3-weeks docetaxel. The authors did not specify whether this was given in the metastatic or adjuvant setting but the cumulative dose reported was high, suggesting a metastatic and prolonged regimen of every-three-weeks docetaxel. A total of 6 of these 10 patients had punctal stenosis and 5 patients had canalicular stenosis. The authors noted that some patients had stenosis at multiple levels, so the true percentage of patients with stenosis was unclear [22].

A more recent study from Japan reporting on excessive tearing as a side effect of every-3-weeks docetaxel for a variety of cancers, most commonly breast cancer but also lung and others, showed a low incidence of excessive tearing of 8% (7/89) after a range of 4–6 cycles of treatment [23]. Approximately 1/3 of these patients were on docetaxel only, but the rest were on a combination of chemotherapy agents with the second and third largest cohorts on docetaxel and cyclophosphamide or trastuzumab plus docetaxel and cyclophosphamide. Six patients were on a regimen involving 5-fluorouracil, which should be noted as also being associated with excessive tearing. All patients who reported excessive tearing had mild symptoms that improved within four months of chemotherapy cessation.

### 4.3. Timing of Onset of Tearing Associated with Docetaxel

Docetaxel-induced excessive tearing typically has a rapid onset regardless of treatment frequency. Mean reported intervals between starting weekly docetaxel therapy and the onset of excessive tearing were two months (range of 1–3 months) in one study, and a median of 2.5 months (range of 1.5–5 months) in another study [8,9].

Similar findings have been noted in the every-3-weeks docetaxel group: onset of excessive tearing was noted around months 2–3, with a range of 1–5 months [8,10]. In patients receiving every-3-weeks docetaxel for short durations of treatment, such as in the adjuvant setting in patients with early breast cancer, excessive tearing is expected to resolve by about 3–4 months after the cessation of chemotherapy [10,23].

### 4.4. Proposed Pathophysiology of Canalicular Stenosis and Excessive Tearing Associated with Docetaxel

Docetaxel-induced excessive tearing is thought to occur through the secretion of docetaxel in the tear film leading to direct contact between the drug and the ocular surface and the tear drainage apparatus. This is supported by the finding that docetaxel was found in tear samples taken within 24 h of administration of the drug, with mean drug concentrations 4.5 higher in tears than serum [4]. In three patients on docetaxel who underwent surgery for excessive tearing, biopsies of the lacrimal sac and nasal mucosa showed marked fibrosis of the stroma and chronic inflammation [25]. No biopsies of the canaliculus were performed in this study, although it is hypothesized that these changes occur throughout the length of the tear drainage system.

A study on rats treated with intraperitoneal docetaxel histologically evaluated multiple ocular tissues postmortem and showed cystic dilation and an inflammatory infiltrate in the lacrimal gland on hematoxylin and eosin staining [26]. There was also an increased presence of inflammatory markers including IL-6 on immunohistochemistry in the lacrimal gland and cornea. Although the tear drainage system was not studied, the authors concluded that the increased inflammation explained many reported ocular side effects of docetaxel including tearing, dry eyes, conjunctivitis, and meibomian gland dysfunction.

As previously mentioned, dry eyes are a key cause of reflex tearing and a major contributor to the excessive tearing reported by patients on docetaxel, especially in patients treated with an every-3-weeks regimen for a short duration in the adjuvant setting. Although this potential cause of “excessive tearing” from docetaxel has been less well studied than canalicular stenosis, docetaxel’s presence in tears has been thought to be an ocular surface irritant inducing reflex tearing. Additionally, two studies have reported meibomian gland dropout and dysfunction in the eyelids of cancer patients on docetaxel and a variety of other chemotherapies [13,14]. The lipid layer of the tear film secreted by the meibomian glands is an important component of the tears and is responsible for preventing the evaporation of tears. Poorly functioning meibomian glands are a common cause of dry eyes and reflex tearing.

A prospective study in breast cancer patients analyzed the meibomian gland changes in detail in 10 patients on docetaxel and 10 patients on other chemotherapy regimens [14]. The authors did not state what comprised these other chemotherapy regimens. The meibomian gland number and quality worsened in patients from pretreatment to 4–6 months into treatment with docetaxel compared to treatment with other chemotherapies, which experienced no change in these parameters over time. The tear break-up time, a measurement that indicated tear film instability and more rapid tear evaporation, also worsened in the patients treated with docetaxel. The last follow-up was two months after stopping docetaxel and by this visit, tearing resolved in most patients after the discontinuation of docetaxel, and the changes in the meibomian glands were reversible as well.

### 4.5. Current Trends in Docetaxel Use

Our group’s publications in the early 2000s that raised awareness of excessive tearing and canalicular stenosis associated with docetaxel were almost exclusively in patients with metastatic breast cancer [3,5,6,7,8,27,28]. Weekly administration of docetaxel was initially explored and attractive due to less severe serious side effects such as myelosuppression, but non-hematologic toxicities, such as nail changes and canalicular stenosis, were more common with weekly administration of docetaxel [20]. Furthermore, weekly docetaxel has been shown to offer no survival benefits compared to every-3-weeks treatment in patients with metastatic breast cancer [29]. Since its first use over 25 years ago, the use of weekly docetaxel in patients with metastatic breast cancer has been largely replaced by weekly paclitaxel. It should be noted that excessive tearing related to paclitaxel use is much less commonly reported [24,30,31,32].

Today, docetaxel is used more commonly in the adjuvant setting in patients with early breast cancer, and the schedule of administration of the drug is in cycles of every 3 weeks. Because of this shift in oncologic treatment trends, it is less common to encounter patients with severe canalicular stenosis associated with docetaxel and thus, surgical intervention is less common. As stated above, most patients with breast cancer who received docetaxel every three weeks for a maximum duration of 4 months in the adjuvant setting only experienced transient excessive tearing and likely had a significant component of dry eye syndrome and reflex tearing. Such patients should be referred to an ophthalmologist and be carefully examined, monitored, and supported throughout their treatment course through conservative medical management such as lubricating drops and the cautious use of topical steroid drops and reassured that symptoms likely will resolve once the short duration of docetaxel treatment is completed.

A recent study published in 2023 analyzed the ophthalmic adverse events of docetaxel and paclitaxel from the United States FDA Adverse Event Reporting System from 2013 through 2021 [33]. This database encompasses adverse events voluntarily reported by a variety of sources, including healthcare workers, patients, lawyers, and pharmaceutical companies. As expected, tearing-related terminology (dacryostenosis, increased lacrimation, lacrimation disorder, and xerophthalmia) were significantly more common in docetaxel patients than paclitaxel patients. While the proportion of reported lacrimal adverse events compared to all adverse events for docetaxel and paclitaxel was largely consistent over the study period, there was a steep uptick in reports in 2021. While the exact reason for this steep increase in reporting in 2021 is not clear, we note that there are websites that specifically solicit litigation for excessive tearing associated with docetaxel starting in 2020 [34].

### 4.6. Treatment of Excessive Tearing Associated with Docetaxel

**Figure 1:**
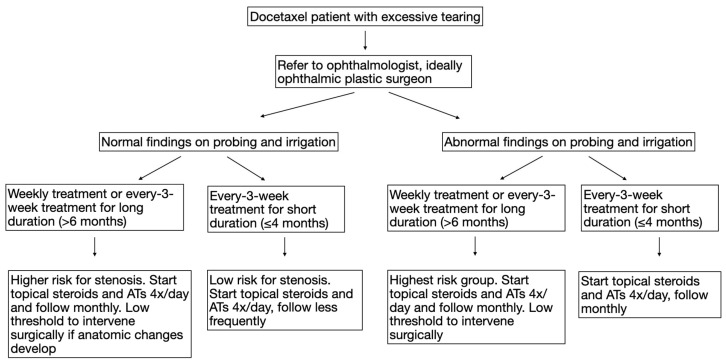
Algorithm for treatment of excessive tearing associated with docetaxel (ATs: artificial tears).

Patients who report excessive tearing associated with docetaxel therapy should be evaluated by an ophthalmologist once they become symptomatic. Timely referral by medical oncologists to an ophthalmic plastic surgeon or an ophthalmologist with an in-depth knowledge of the lacrimal drainage apparatus is important. An algorithm summarizing treatment recommendations for patients who have excessive tearing associated with docetaxel is shown in Figure 1. Patients should undergo a full ophthalmic exam as described previously in this review, specifically including probing and irrigation. We recommend starting all patients with symptomatic excessive tearing associated with docetaxel on topical steroid therapy. The clinician may choose their preferred topical steroid, but we tend to use tobramycin 0.3%–dexamethasone 0.1% four times per day because the antibiotic prophylaxes against infection in these patients, who may be neutropenic. Prednisolone acetate 1% is also a reasonable option. Patients on topical steroids should be monitored by an ophthalmologist, as there is a significant risk of ocular toxicity associated with this class of drugs. Frequent artificial tear use can also be helpful, as it not only soothes the ocular surface and helps with dry eye symptoms but can also dilute the drug concentration in tears. One study randomized patients on weekly docetaxel therapy to topical artificial tears six times per day versus topical steroids six times per day (dexamethasone 0.1%) [18]. At nine weeks, an equal proportion of patients in each group had developed canalicular stenosis (9/20, 45%), and thus, the authors concluded that steroid drops were not superior to artificial tears and recommended prophylactic artificial tears throughout the course of docetaxel therapy. It is important to note that the beneficial effects of topical steroids are expected to be more significant in cases of transient mild excessive tearing in patients who receive every-3-weeks docetaxel for short durations of treatment rather than the higher-risk patient population on weekly treatment studied by these authors.

In patients who develop stenosis, surgery should be considered after a trial period of topical steroids. If the patient is not seeing an ophthalmic plastic surgeon when anatomic abnormalities are detected on probing and irrigation, prompt referral is recommended. More severe or worsening stenosis in higher-risk patients should be surgically addressed more urgently. The ophthalmic plastic surgeon should recommend the appropriate surgical intervention based on the location and severity of stenosis: punctoplasty for punctal stenosis, bicanalicular silicone stenting of the nasolacrimal duct with canalicular dilation or other adjuncts for canalicular stenosis, conjunctivo-DCR (c-DCR) for frank canalicular obstruction, and DCR for canalicular stenosis too severe for stenting alone. In patients who are on weekly docetaxel or in those on an every-three-weeks regimen for longer than 6 months (in a metastatic setting), silicone stenting may be considered to prevent complete blockage of the canaliculi. Once a patient has had silicone stenting, the stents should be kept in place for the entire duration of exposure to docetaxel and at least three months after docetaxel treatment is discontinued [7,28].

In patients on docetaxel who required surgery, surgery is reported as quite successful [7,8,22,35]. In one study, in 71 patients with metastatic breast cancer on weekly therapy, 30 patients (59 eyes) underwent surgery and twenty-nine of these patients (97%) reported total resolution of or improvement in excessive tearing and had patent tear drainage systems at a median follow-up of 5.5 months [7]. Surgeries were as follows: 23 patients (39 eyes) underwent bicanalicular silicone stenting, 9 patients (13 eyes) DCR, and 4 patients (7 eyes) c-DCR. The one patient with persistent tearing had scarring around the silicone stent after DCR and would have benefited from c-DCR but declined additional surgery. In this cohort of 71 patients on weekly treatment, there were an additional 21 patients for whom surgery was recommended, but they declined.

Another study that reported on surgical outcomes in six patients with metastatic breast cancer on weekly docetaxel noted that all patients (100%) had complete resolution of excessive tearing postoperatively [8]. Two underwent silicone stent placement, three bilateral DCR, and one unilateral DCR and contralateral stent placement.

In Kang et al.’s report of surgical outcomes in patients with multiple etiologies of cancer-associated excessive tearing, 10 patients had been treated with every-3-weeks docetaxel [22]. The authors reported a 100% success rate in patients who had either punctoplasty or silicone stenting.

In summary, patients exposed to short-term (less than or equal to 4 months) therapy with docetaxel administered every three weeks, typically early breast cancer patients receiving docetaxel in the adjuvant setting, are not at a significant risk of punctal or canalicular stenosis. These patients should not require surgical intervention [28]. If these low-risk patients develop excessive tearing, examination with probing and irrigation should be done, and judicious use of topical steroids is successful in preventing most cases of stenosis. Follow-up visits may be less frequent, and most symptoms will resolve with topical treatments and time after the cessation of chemotherapy. Patients should be reassured.

In patients with planned prolonged courses of every-3-weeks docetaxel (typically greater than 6 months), for example, patients with metastatic breast cancer who may receive every-3-weeks docetaxel for several years, closer follow-up is appropriate and silicone stenting should be considered if progressive canalicular stenosis is detected. As mentioned previously, weekly administration of docetaxel in patients with breast cancer is of mostly historical significance; however, if such patients are encountered in practice today, they should be considered high risk for the development of punctal, canalicular and lacrimal duct stenosis. Such patients need to be monitored at least monthly by an ophthalmologist with expertise in lacrimal disorders. If excessive tearing as a symptom and canalicular stenosis as a physical exam finding worsen as treatment with weekly docetaxel continues, bicanalicular silicone stenting should be recommended to prevent further canalicular stenosis. Early intervention in this setting is important to prevent complete canalicular obstruction necessitating more invasive surgery [35]. If stenosis develops in other parts of the tear drainage system, similar early intervention is recommended as we expect that the anatomic findings will worsen with continued weekly docetaxel therapy.

## 5. Conclusions

The knowledge and understanding of excessive tearing as a possible ocular side effect of docetaxel is important to enable prompt recognition and referral to prevent permanent anatomic changes in the lacrimal drainage apparatus in patients on weekly docetaxel or in patients who receive docetaxel every three weeks for extended periods (longer than 6 months). These two groups of patients are may require surgical intervention. The late referral of such patients is problematic, as early intervention is essential.

In contrast, in patients who receive docetaxel every three weeks in the adjuvant setting for early breast cancer, the anatomic findings of punctal, canalicular, and lacrimal duct stenosis are rare. Many of these patients, however, have symptomatic excessive tearing due to the secretion of docetaxel in the tear film and secondary ocular irritation or dry eye syndrome, which can be hormonally induced, from meibomian gland changes or from a multitude of other etiologies. In such cases, the prompt referral of symptomatic patients to an ophthalmologist is still important, but it is also important to recognize that most of such patients respond to conservative management with probing and irrigation and judicious use of topical steroids. Hasty and unnecessary surgical intervention in such patients can lead to unhappy patients and increased morbidity.

## Data Availability

No new data were created or analyzed in this study. Data sharing is not applicable to this article.

## References

[1] Bicer T., Imamoglu G.I., Dogan A.S., Avarisli N.A., Kabatas N., Bicer B.K., Gurdal C. (2020). The effects of adjuvant hormonotherapy on tear functions in patients with breast cancer. Int. Ophthalmol..

[2] Grasso A., Salerno A., Micera A., Ferraro A., Fornaro E., Esposito G., Coassin M., Altomare V., Di Zazzo A. (2025). Secondary dry eye disease in breast cancer patients: A pilot study. Breast.

[3] Esmaeli B., Valero V., Ahmadi M., Booser D. (2001). Canalicular stenosis secondary to docetaxel (taxotere). Ophthalmology.

[4] Esmaeli B., Ahmadi M.A., Rivera E., Valero V., Hutto T., Jackson D.M., Newman R.A. (2002). Docetaxel secretion in tears: Association with lacrimal drainage obstruction. Arch. Ophthalmol..

[5] Esmaeli B., Hortobagyi G.N., Esteva F.J., Booser D., Ahmadi M., Rivera E., Arbuckle R., Delpassand E., Guerra L., Valero V. (2002). Canalicular stenosis secondary to weekly versus every-3-weeks docetaxel in patients with metastatic breast cancer. Ophthalmology.

[6] Esmaeli B., Hortobagyi G., Esteva F., Valero V., Ahmadi M.A., Booser D., Ibrahim N., Delpassand E., Arbuckle R. (2002). Canalicular stenosis secondary to weekly docetaxel: A potentially preventable side effect. Ann. Oncol..

[7] Esmaeli B., Hidaji L., Adinin R.B., Faustina M., Coats C., Arbuckle R., Rivera E., Valero V., Tu S., Ahmadi M.A. (2003). Blockage of the lacrimal drainage apparatus as a side effect of docetaxel therapy. Cancer.

[8] Esmaeli B., Amin S., Valero V., Adinin R., Arbuckle R., Banay R., Do K.-A., Rivera E. (2006). Prospective Study of Incidence and Severity of Epiphora and Canalicular Stenosis in Patients with Metastatic Breast Cancer Receiving Docetaxel. J. Clin. Oncol..

[9] Tsalic M., Gilboa M., Visel B., Miller B., Haim N. (2006). Epiphora (Excessive Tearing) and Other Ocular Manifestations Related to Weekly Docetaxel: Underestimated Dose-Limiting Toxicity. Med. Oncol..

[10] Chan A., Su C., de Boer R.H., Gajdatsy A. (2013). Prevalence of Excessive Tearing in Women with Early Breast Cancer Receiving Adjuvant Docetaxel-Based Chemotherapy. J. Clin. Oncol..

[11] Esmaeli B., Valero V. (2013). Epiphora and Canalicular Stenosis Associated with Adjuvant Docetaxel in Early Breast Cancer: Is Excessive Tearing Clinically Important?. J. Clin. Oncol..

[12] Mansur C., Pfeiffer M.L., Esmaeli B. (2017). Evaluation and Management of Chemotherapy-Induced Epiphora, Punctal and Canalicular Stenosis, and Nasolacrimal Duct Obstruction. Ophthalmic Plast. Reconstr. Surg..

[13] Lee H., Yoon S., Baek S. (2023). Causes of Tearing in Patients with Chemotherapy: Meibomian Gland Dysfunction Versus Lacrimal Drainage Obstruction. J. Craniofacial Surg..

[14] Stoicescu E.A., Cherecheanu A.P. (2023). Meibomian gland changes in breast cancer patients treated with docetaxel-partial results. Rom. J. Ophthalmol..

[15] Esteva F.J., Valero V., Booser D., Guerra L.T., Murray J.L., Pusztai L., Cristofanilli M., Arun B., Esmaeli B., Fritsche H.A. (2002). Phase II Study of Weekly Docetaxel and Trastuzumab for Patients With HER-2–Overexpressing Metastatic Breast Cancer. J. Clin. Oncol..

[16] Pouyeh B., Viteri E., Feuer W., Lee D.J., Florez H., Fabian J.A., Perez V.L., Galor A. (2012). Impact of Ocular Surface Symptoms on Quality of Life in a United States Veterans Affairs Population. Arch. Ophthalmol..

[17] Kintzel P.E., Michaud L.B., Lange M.K. (2006). Docetaxel-Associated Epiphora. Pharmacother. J. Hum. Pharmacol. Drug Ther..

[18] Leyssens B., Wildiers H., Lobelle J.P., Gillis A., Paridaens R., Mombaerts I. (2009). A double-blind randomized phase II study on the efficacy of topical eye treatment in the prevention of docetaxel-induced dacryostenosis. Ann. Oncol..

[19] Drugs@FDA: FDA-Approved Drugs. https://www.fda.gov/drugsatfda.

[20] van Eijk M., Vermunt M.A.C., van Werkhoven E., Wilthagen E.A., Huitema A.D.R., Beijnen J.H. (2022). The influence of docetaxel schedule on treatment tolerability and efficacy in patients with metastatic breast cancer: A systematic review and meta-analysis of randomized controlled trials. BMC Cancer.

[21] Noguchi Y., Kawashima Y., Kawara H., Tokuyama Y., Tamura Y., Uchiyama K., Shimizu Y. (2016). A Retrospective Analysis of Epiphora Due to Docetaxel. Cancer Chemother..

[22] Kang S., Seo J.W., Sa H.-S. (2017). Cancer-associated epiphora: A retrospective analysis of referrals to a tertiary oculoplastic practice. Br. J. Ophthalmol..

[23] Noguchi Y., Kawashima Y., Maruyama M., Kawara H., Tokuyama Y., Uchiyama K., Shimizu Y. (2019). Current status of eye disorders caused by docetaxel administration every 3 weeks: A case-control study in Japanese patients. J. Oncol. Pharm. Pr..

[24] Iwai C., Miyawaki A., Konishi T., Okada A., Fujita A., Jo T., Yasunaga H. (2025). Ocular adverse events of perioperative adjuvant docetaxel vs paclitaxel for breast cancer: Propensity-score overlap-weighted analysis. Breast Cancer Res. Treat..

[25] Esmaeli B., Burnstine M.A., Ahmadi M.A., Prieto V.G. (2003). Docetaxel-Induced Histologic Changes in the Lacrimal Sac and the Nasal Mucosa. Ophthalmic Plast. Reconstr. Surg..

[26] Unsal A.I.A., Kahyaoglu F., Eroglu S.A., Onal T., Demir B., Barutca S., Demirci B. (2025). An Experimental Study on the Widely Used Chemotherapeutic Docetaxel: Does It Induce Inflammation, Ischemia, and Neurodegeneration in the Eye, Causing Dry Eye and Blurred Vision in a Real-Life Scenario?. Curr. Eye Res..

[27] Esmaeli B., Hortobagyi G.N. (2001). Canalicular Stenosis as the Underlying Mechanism for Epiphora in Patients Receiving Weekly Docetaxel. Oncologist.

[28] Esmaeli B. (2005). Management of Excessive Tearing as a Side Effect of Docetaxel. Clin. Breast Cancer.

[29] Schröder C.P., de Munck L., Westermann A.M., Smit W.M., Creemers G.-J.M., de Graaf H., Stouthard J.M., van Deijk G., Erjavec Z., van Bochove A. (2011). Weekly docetaxel in metastatic breast cancer patients: No superior benefits compared to three-weekly docetaxel. Eur. J. Cancer.

[30] McCartney E., Valluri S., Rushing D., Burgett R. (2007). Upper and Lower System Nasolacrimal Duct Stenosis Secondary to Paclitaxel. Ophthalmic Plast. Reconstr. Surg..

[31] Savar A.M., Esmaeli B.M. (2009). Re: “Upper and Lower System Nasolacrimal Duct Stenosis Secondary to Paclitaxel”. Ophthalmic Plast. Reconstr. Surg..

[32] Fortes B.H., Liou H., Dalvin L.A. (2020). Ophthalmic adverse effects of taxanes: The Mayo Clinic experience. Eur. J. Ophthalmol..

[33] McGwin G., Contorno T., Vicinanzo M.G., Owsley C. (2023). The Association Between Taxane Use and Lacrimal Disorders. Curr. Eye Res..

[34] Taxotere (Docetaxel) Eye Injuires Lawsuit. https://paulllp.com/mass-torts/taxotere-eye-injuries-lawsuit/.

[35] Ahmadi M.A., Esmaeli B. (2001). Surgical Treatment of Canalicular Stenosis in Patients Receiving Docetaxel Weekly. Arch. Ophthalmol..

